# LC3/FtMt Colocalization Patterns Reveal the Progression of FtMt Accumulation in Nigral Neurons of Patients with Progressive Supranuclear Palsy

**DOI:** 10.3390/ijms23010537

**Published:** 2022-01-04

**Authors:** Zulzikry Hafiz Abu Bakar, Jean-Pierre Bellier, Daijiro Yanagisawa, Tomoko Kato, Ken-ichi Mukaisho, Ikuo Tooyama

**Affiliations:** 1 Molecular Neuroscience Research Center, Shiga University of Medical Science, Seta Tsukinowa-cho, Otsu 520-2192, Japan; zikry@belle.shiga-med.ac.jp (Z.H.A.B.); daijiroy@belle.shiga-med.ac.jp (D.Y.); tkato@belle.shiga-med.ac.jp (T.K.); 2 Department of Pathology of Pathology, Shiga University of Medical Science, Seta Tsukinowa-cho, Otsu 520-2192, Japan; mukaisho@belle.shiga-med.ac.jp; 3 Education Center for Medicine and Nursing, Shiga University of Medical Science, Seta Tsukinowa-cho, Otsu 520-2192, Japan

**Keywords:** progressive supranuclear palsy, mitochondrial ferritin, FtMt, LC3, mitophagy, midbrain

## Abstract

Mitochondrial ferritin (FtMt) is a mitochondrial iron storage protein associated with neurodegenerative diseases. In patients with progressive supranuclear palsy (PSP), FtMt was shown to accumulate in nigral neurons. Here, we investigated FtMt and LC3 in the post-mortem midbrain of PSP patients to reveal novel aspects of the pathology. Immunohistochemistry was used to assess the distribution and abnormal changes in FtMt and LC3 immunoreactivities. Colocalization analysis using double immunofluorescence was performed, and subcellular patterns were examined using 3D imaging and modeling. In the substantia nigra pars compacta (SNc), strong FtMt-IR and LC3-IR were observed in the neurons of PSP patients. In other midbrain regions, such as the superior colliculus, the FtMt-IR and LC3-IR remained unchanged. In the SNc, nigral neurons were categorized into four patterns based on subcellular LC3/FtMt immunofluorescence intensities, degree of colocalization, and subcellular overlapping. This categorization suggested that concomitant accumulation of LC3/FtMt is related to mitophagy processes. Using the LC3-IR to stage neuronal damage, we retraced LC3/FtMt patterns and revealed the progression of FtMt accumulation in nigral neurons. Informed by these findings, we proposed a hypothesis to explain the function of FtMt during PSP progression.

## 1. Introduction

Progressive supranuclear palsy (PSP) is a rare neurodegenerative disorder with 3–4 cases per million people reported annually. Like Parkinson’s disease, the pathological features of PSP include loss of nigral neurons [1]. PSP accounts for approximately 5% of Parkinson’s disease incidence [2] and is the most common form of atypical parkinsonism [3]. PSP cases are predicted to increase from 2030 [4]; however, accurate diagnosis remains difficult and a cure is yet to be found [5]. Although scarcely described, PSP is a tauopathy with considerable accumulation of four-repeat tau and formation of aberrant tau structures (neurofibril tangles, globose-type tangles, fine threads, and small dot structures) appearing as additional major pathological features [6,7]. Mitochondrial dysfunction is frequently associated with PSP [8,9] and could be used to leverage the development of novel diagnostic methods.

Mitochondrial ferritin (FtMt) is a protein that sequesters and stores iron in the mitochondria [10]. It plays roles in iron homeostasis, availability, and transport [11,12,13]. Dysregulation of FtMt expression was associated with neurodegenerative diseases [14]. In a previous study, we found the accumulation of FtMt in the post-mortem midbrains of PSP patients [6]. In addition, increased FtMt expression was associated with cellular damage. For instance, we observed increased FtMt expression in human neuroblastoma cells (IMR-32) treated with proinflammatory cytokines [15], while other researchers found increased FtMt expression in SH-SY5Y treated with hydrogen peroxide [16].

In neurodegenerative disorders, such as Alzheimer’s disease, pTau accumulation induces mitochondria abnormalities [17] and defective mitophagy [18], which lead to excessive mitochondrial fragmentation [19]. We previously observed the accumulation of pTau in the midbrain of PSP patients colocalizing with FtMt [6]. Interestingly, FtMt-induced mitophagy in FtMt-overexpressing ARPE-19 cells was reported [20]. LC3, a marker for autophagosomes, is extensively used to determine mitophagy status [21,22]. Here, we investigated FtMt and LC3 in the post-mortem midbrain of control and PSP cases to reveal new aspects of the related pathology. Our experimental design was based on a straightforward immunohistochemical study of LC3 and FtMt using antibodies that are well-characterized and validated for use in human tissues, including PSP patients’ tissues [6,23]. Single immunohistochemical staining using DAB was used to determine whether FtMt and LC3 changes occurred in the midbrains of PSP patients. Double immunofluorescence staining was performed to further confirm observations and assess colocalization. Lastly, 3D image reconstruction and modeling were carried out to examine the spatial arrangement of FtMt and LC3 at the subcellular level.

## 2. Results

### 2.1. FtMt and LC3 Immunoreactivity in the Midbrain of Control and PSP Cases

Single immunostaining for FtMt and LC3 were performed on paraffin-embedded sections of midbrains from control and PSP patient cases. Here, we describe the results in two regions: the substantia nigra pars compacta (SNc) and superior colliculus (SC).

**Figure 1:**
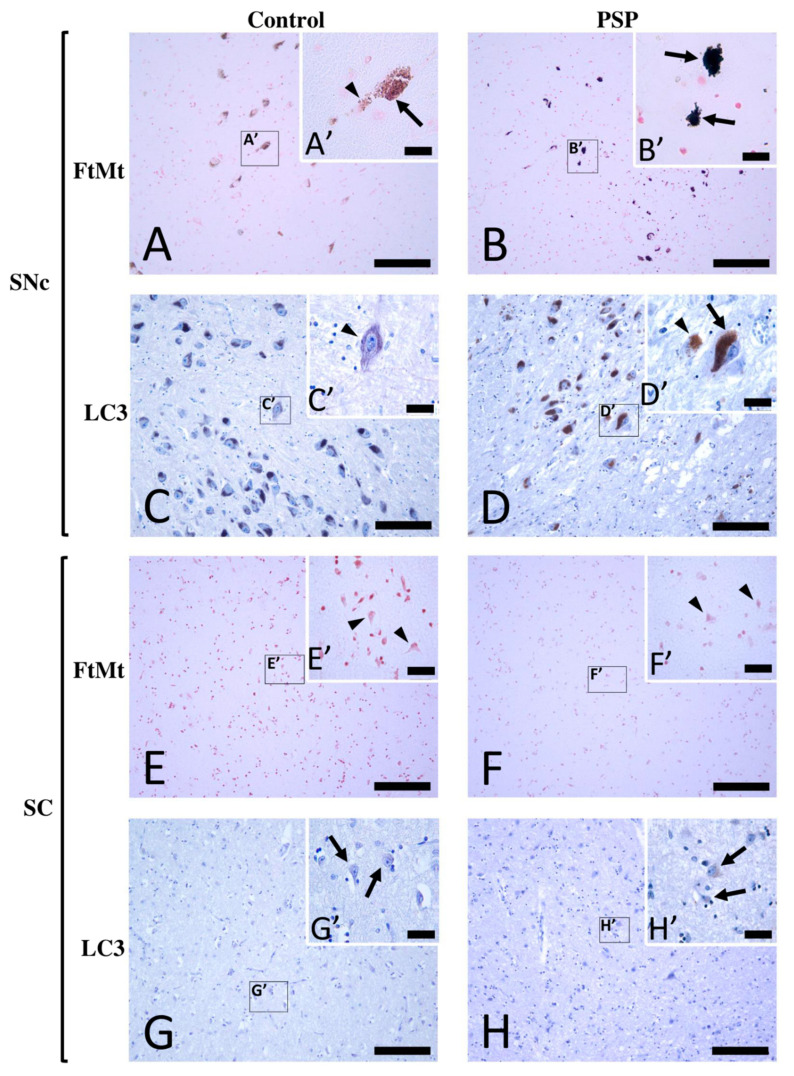
Immunohistochemistry for FtMt and LC3 in the SNc and SC of a control case (control-2) and a PSP patient case (PSP-4). (**A**) FtMt-IR in a control case’s SNc and (**A′**) high-magnification image of a neuromelanin-containing neuron in boxed area in (**A**); the arrowhead indicates weak brown dotty neuromelanin deposits, while the arrow indicates puncta with strong dark FtMt-IR. (**B**) FtMt-IR in a PSP case’s SNc and (**B′**) high-magnification image of the boxed area in (**B**); arrows indicate neurons filled with puncta showing intense dark FtMt-IR. (**C**) LC3-IR in a control case’s SNc and (**C′**) high-magnification image of the boxed area in (**C**); the arrowhead indicates weak LC3-IR in a neuron devoid of neuromelanin. (**D**) LC3-IR in a PSP case’s SNc and (**D′**) high-magnification image of the boxed area in (**D**); the arrow and arrowhead indicate LC3-immunoreactive neuromelanin-containing neurons and LC3-immunoreactive neurons devoid of neuromelanin, respectively. (**E**) IHC for FtMt in the SC of a control case revealed no staining and (**E′**) high-magnification image of the boxed area in (**E**); arrowheads point to neurons, none of which showed FtMt staining. (**F**) IHC for FtMt in a PSP case’s SC that revealed no staining and (**F′**) high-magnification image of the boxed area in (**F**); arrowheads point to neurons, none of which showed FtMt staining. (**G**) LC3-IR in the SC of a control case and (**G′**) high-magnification image of the boxed area in (**G**); the arrow indicates a neuron with LC3-IR in the soma. (**H**) LC3-IR in the SC of a PSP case and (**H′**) high-magnification image of the boxed area in (**H**); arrows indicate neurons with LC3-IR in the soma. All sections stained with FtMt were counterstained with neutral red stain. All sections stained with LC3 were counterstained with hematoxylin. Scale bars: 100 µm (**A**–**H**) and 20 µm (**A′**–**H′**).

In the SNcs of the control cases, weak-to-moderate FtMt immunoreactivity (FtMt-IR) was preferentially observed in neurons (Figure 1A). At high magnification, small dark purple FtMt immunoreactive puncta (arrows in Figure 1A′) were distinct from neuromelanin dotty brownish deposits (arrowheads in Figure 1A′). Weak LC3 immunoreactivity (LC3-IR) was preferentially found in the SNc neurons (Figure 1C). At high magnification, LC3-IR could be observed in the soma of neurons with little or no neuromelanin contents (arrowheads in Figure 1C′). However, due to its weak intensity, the LC3-IR remained ambiguous in neurons with high neuromelanin contents. In the SCs of the control cases, there was no apparent FtMt-IR (Figure 1E). High-magnification observation confirmed that the neurons were devoid of FtMt-IR (arrowheads in Figure 1E′). Faint LC3-IR was anecdotally observed in a few neurons (Figure 1G,G′).

In the SNcs of the PSP cases, strong-to-intense FtMt-IR was observed (Figure 1B). FtMt-IR was localized in the neurons (arrows in Figure 1B’), and many FtMt immunoreactive dots were observed in the extracellular brain matrix (Figure 1B’). Strong LC3-IR was observed in most neurons (Figure 1D). Since the LC3-IR was intense, it was detectable not only in neurons with low neuromelanin contents but also in those with high contents (arrows in Figure 1D′). The SC of a PSP case was devoid of FtMt-IR (Figure 1F,F′). As in the control cases, a few neurons with faint LC3-IR were observed in the SC (Figure 1G,G′). The SC of PSP patients anecdotally showed a few neurons with faint LC3-IR (Figure 1H,H′). These observations indicated the accumulation of FtMt and LC3 immunoreactivities in the SNcs, but not in the SCs, of the PSP patients.

### 2.2. FtMt and LC3 Colocalization in the Substantia Nigra of Control and PSP Cases

**Figure 2:**
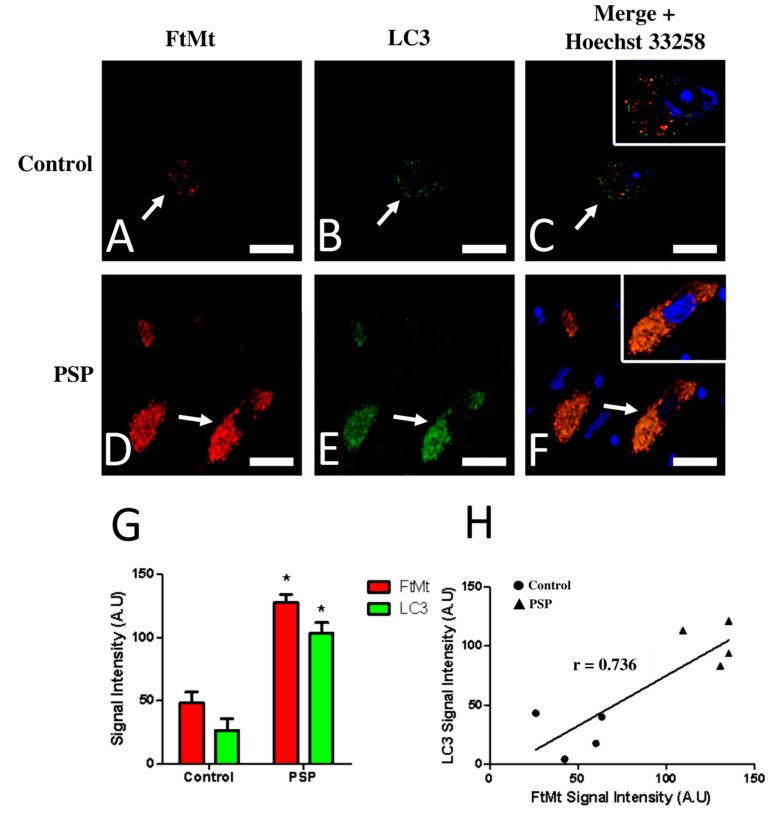
Confocal imaging (**A**–**F**) and quantitative analysis (**G**,**H**) of double immunofluorescence histochemistry for FtMt and LC3 in the SNc of a control case (control-1) and a PSP patient (PSP-4). Immunoreactive signal intensity of the control and PSP cases were quantified from 5 stacked images. (**A**) FtMt-IR in a control case’s SNc. The arrow indicates a neuron with weak FtMt-IR; staining was limited to puncta. (**B**) LC3-IR in a control case’s SNc. The arrow indicates a neuron showing weak LC3-IR; staining was limited to puncta. (**C**) Merged images (**A**,**B**). (**D**) FtMt-IR in a PSP case’s SNc. The arrow points at a neuron with strong FtMt-IR. (**E**) LC3-IR in a PSP case’s SNc. The arrow points at a neuron with strong FtMt-IR. (**F**) Merged images (**D**,**E**). Panels (**C**,**F**) contain an additional inset with vivid nuclei staining to illustrate the boundaries of the cell nucleus (magnification is the same as the main panel). (**G**) Quantitative signal intensity analysis for the FtMt-IR and LC3-IR in the SNcs of control and PSP cases. * indicates a significant difference (*p* < 0.05) compared to the control cases. Data are presented as the mean ± standard error of the mean (SEM). (**H**) Plotted values of FtMt and LC3 immunofluorescence intensities. The coefficient of correlation (r) is indicated on the graph. Nuclei stained with Hoechst 33258 are shown in the merged images. All scale bars: 20 µm.

Double immunofluorescence followed by confocal microscopy analysis was performed to further characterize and quantitatively estimate the LC3-IR and FtMt-IR in the substantia nigras of the control and PSP cases. The results of the confocal microscope analysis were largely consistent with previous observations in sections revealed with diaminobenzidine. In the SNcs of the control cases (Figure 2A,B), weak-to-moderate FtMt-IR and LC3-IR were localized in the soma of neurons. In the SNcs of the PSP cases (Figure 2D,E), intense FtMt-IR and LC3-IR were also localized in the soma of neurons. Notably, the LC3-IR alone (without FtMt-IR) was not observed, even anecdotally, in the PSP or control cases. Confocal microscopy analysis confirmed that the LC3-IR and FtMt-IR appeared as small puncta in the soma of nigral neurons. Quantitative analysis of fluorescence signal intensities showed a significant 2.5-fold FtMt-IR increase (*p* = 0.028) and a significant threefold LC3-IR increase (*p* = 0.029) in the SNcs of the PSP patients (Figure 2G). Correlation analysis for the FtMt-IR and LC3-IR intensities in the control and PSP cases’ SNcs showed a value higher than 0.73, indicating either a tendency for the FtMt-IR and LC3-IR to increase concomitantly in all neurons or subpopulations of neurons following different trends.

Confocal microscope observations using a 0.46 mm thick focal plane were sufficient to accurately assess the degree of colocalization between the FtMt-IR and LC3-IR. Colocalization of FtMt-IR and LC3-IR was rare in the SNcs of the control cases (Figure 2C) but was frequent in the SNcs of the PSP patients (Figure 2F). The Pearson colocalization coefficient (PCC) for the FtMt-IR and LC3-IR was calculated in the SNc of each PSP and control case (Table 1). In most control cases (control-2, control-3, and control-4), The PCC values for FtMt-IR and LC3-IR were inferior to 0.42, indicating a rather low degree of colocalization. One control case (control-1) showed an equivocal PCC value of 0.539. The PCC values for the FtMt-IR and LC3-IR in the PSP cases were slightly superior (0.62) to those of the controls, indicating a weak degree of colocalization and suggesting neuronal subpopulations with heterogeneous degrees of colocalization for the FtMt and LC3.

### 2.3. Subpopulations of FtMt-IR Neurons in SNcs of PSP Patients

**Figure 3:**
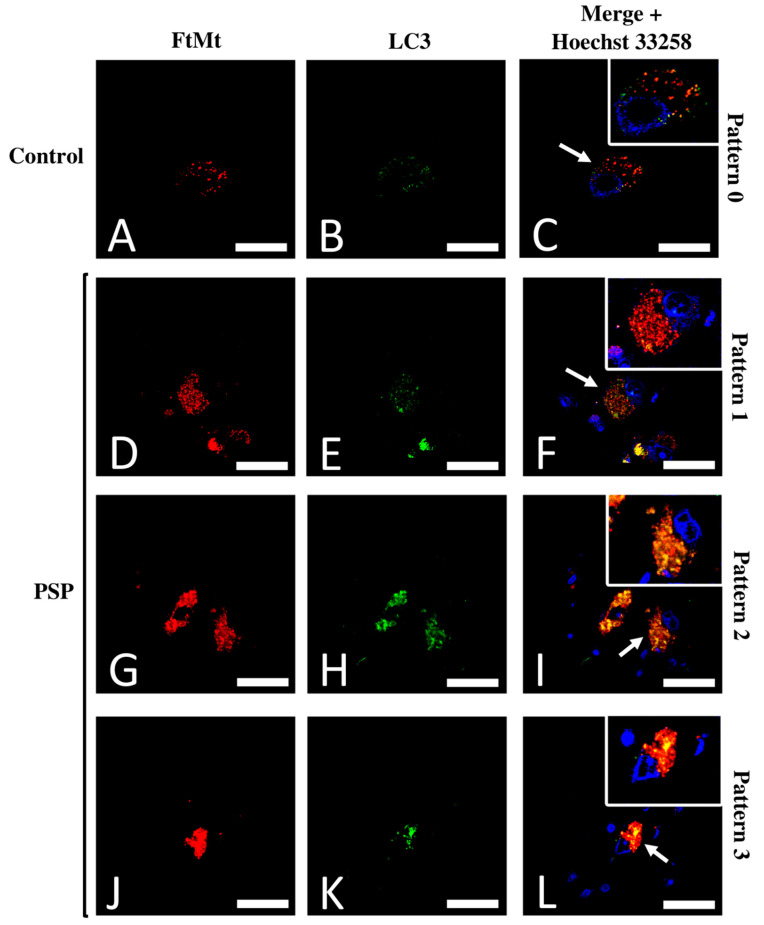
Typical patterns of the FtMt-IR and LC3-IR colocalization. (**A**) FtMt-IR in a control case’s SNc. (**B**) LC3-IR in a control case’s SNc. (**C**) Merged images (**A**,**B**); the arrow points to a neuron with a typical “pattern 0.” (**D**) FtMt-IR in a PSP case’s SNc. (**E**) The LC3-IR in a PSP case’s SNc. (**F**) Merged images (**D**,**E**); the arrow points to a neuron with a typical “pattern 1.” (**G**) FtMt-IR in a PSP case’s SNc. (**H**) LC3-IR in a PSP case’s SNc. (**I**) Merged images **G** and **H**; the arrow points to a neuron with a typical “pattern 2.” (**J**) FtMt-IR in a PSP case’s SNc. (**K**) LC3-IR in a PSP case’s SNc. (**L**) Merged images (**J**,**K**); the arrow points to a neuron with a typical “pattern 3.” Panels (**C**,**F**,**I**,**L**) contain an additional inset with vivid nuclei staining to illustrate the boundaries of the cell nucleus (magnification is the same as the main panel). Pictures in (**A**–**C**) were from the control-1 case. Pictures in (**D**–**F**) were from the PSP-3 case. Pictures in (**G**–**I**) were from the PSP-4 case. Pictures in (**J**–**L**) were from the PSP-2 case. Nuclei stained with Hoechst 33258 are shown in the merged images. All scale bars: 20 µm.

Recurrent FtMt-IR and LC3-IR patterns were investigated to identify possible neuronal subpopulations. In control cases, the labeled SNc neurons displayed a homogeneous staining pattern in which the main characteristics were weak-to-moderate scattered FtMt-IR and LC3-IR with a low degree of colocalization (pattern 0, Figure 3A–C). In the PSP cases, the SNc neurons immunolabeled for FtMt and LC3 showed varied staining characteristics that could be roughly categorized into three patterns: The first pattern showed numerous puncta with strong FtMt-IR, fewer puncta with weak-to-moderate LC3-IR, and a limited degree of colocalization between both (pattern 1, Figure 3D–F). The second pattern showed numerous puncta with strong FtMt-IR and LC3-IR with a high degree of colocalization between both (pattern 2, Figure 3G–I). The third pattern showed packed intense FtMt-IR and numerous puncta with strong LC3-IR with a high degree of colocalization between both (pattern 3, Figure 3J–L). The FtMt-IR, LC3-IR, and the degree of colocalization for all patterns are summarized in the first three columns of Table 2.

### 2.4. Assessment of FtMt-IR and LC3-IR Subcellular Arrangement Using 3D Imaging

**Figure 4:**
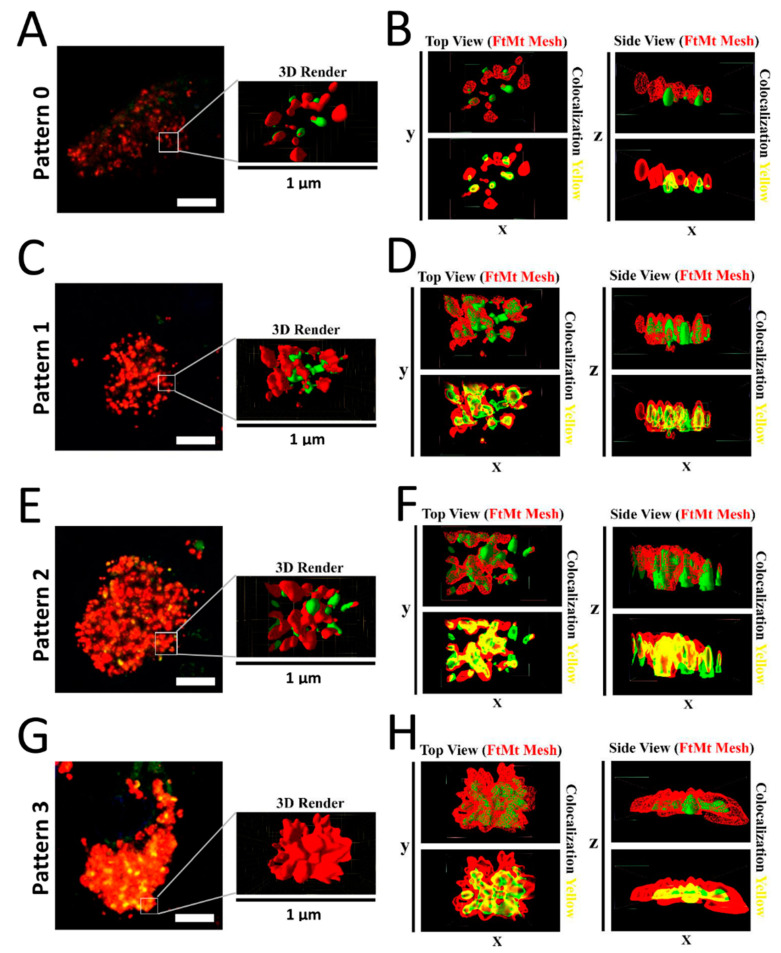
(**A**) Representative image of “pattern 0” (control-3 case). The 3D-rendered surfaces are shown on the right. (**B**) Top and side views of the rendered surface in A. Colocalization of FtMt-IR and LC3-IR was rare. (**C**) Representative image of “pattern 1” (PSP-1 case). The 3D-rendered surfaces are shown on the right. (**D**) Top and side views of the rendered surfaces in C. The FtMt-IR and LC3-IR were overlapping. (**E**) Representative image of “pattern 2” (PSP-1 case). The 3D-rendered surfaces are shown on the right. (**F**) Top and side views of rendered surfaces in E. The FtMt-IR and LC3-IR were overlapping. (**G**) Representative image of “pattern 3” (PSP-1 case). The 3D-rendered surfaces are shown on the right. (**H**) Top and side views of rendered surfaces in G. The LC3-IR was concealed by the FtMt-IR.

Spatial analysis of the subcellular arrangement of the FtMt-IR and LC3-IR was further performed using 3D modeling. These 3D models could be easily rendered and shaded to facilitate the visualization of concealed immunofluorescent signals in merged images. Z-stacked merged images of the FtMt and LC3 immunofluorescence were used to render subcellular scale 3D models for the four types of patterns described above (Figure 4). The analysis of the modeled “pattern 0” (Figure 4A,B) showed no or few overlaps of the FtMt-IR and LC3-IR in subcellular structures. The analysis of the modeled “pattern 1” and “pattern 2” (Figure 4C–F) showed that LC3-IR mostly overlapped with the FtMt-IR. In the analysis of the modeled “pattern 3” (Figure 4G,H), the LC3-IR was enclosed by the FtMt-IR. These features are summarized in the last column of Table 2.

## 3. Discussion

In a previous study of post-mortem midbrains of PSP patients, we found coinciding accumulation of FtMt and pTau in neurons of the substantia nigra [6]. Here, we report the abnormal accumulation of LC3, an autophagosomal marker, in the same neurons. Although this is the first detailed report on the accumulation of LC3 in nigral neurons of PSP patients, our observations are consistent with previous reports describing LC3 and other autophagosomal marker accumulation in the substantia nigra of animal models of neurodegenerative diseases [24,25].

In patients with PSP and other tauopathies, LC3 and pTau accumulation occur simultaneously in the neurons of the frontal cortex [23,26]. As stated above, we previously observed pTau accumulation in the SNc and SC neurons of PSP patients [6]. Here, although we found abnormal accumulation of LC3 in the SNc neurons of PSP patients, LC3 expression in the SC neurons of the PSP cases showed no abnormality. This surprising observation might be the result of different pTau degradation processes. In vitro studies using cultivated neuronal cells showed that tau/pTau degradation processing used both autophagy–lysosomal and ubiquitin–protease pathways [27,28]. Alternatively, the autophagy–lysosomal pathway may cause the accumulation of tau fragments prone to aggregation [29]. In vivo, pTau degradation processing is still unclear. However, our observations indicate that pTau processing in the SC and SNc may involve different degradation systems, as previously suggested [30].

Here, we observed the colocalization of LC3 and FtMt accumulations in the SNc neurons of PSP patient cases. Since FtMt is located in mitochondria [11], this strongly suggests that LC3 is also located and accumulates in the mitochondria of SNc neurons of PSP patients. We also observed LC3-IR in puncta, which might represent the vesicular membrane-bound isoform, namely, LC3-II, which triggers the early steps of autophagy [31]. As the autophagy–lysosomal pathway is involved in FtMt degradation processing, it is naturally tempting to associate LC3/FtMt accumulation in the SNc neurons of PSP patients with mitophagy. Mitophagy is a particular type of autophagy that is related to the selective removal of damaged mitochondria, where LC3 also plays a central role [32]. This interpretation is supported by a previous finding showing that the overexpression of FtMt in human ARPE-19 cells induces the elevation of LC3 expression and triggers mitophagy [20].

**Figure 5:**
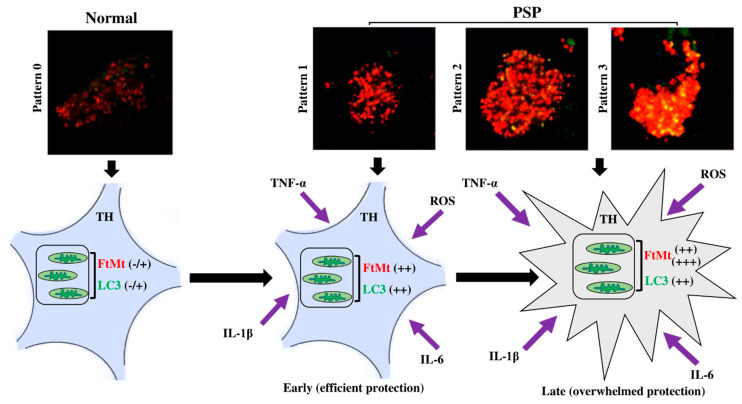
FtMt progression staging and proposed hypothesis for the evolution of function of FtMt, from protective in the early stage of the disease to being overwhelmed in the late stage.

In the present study, we categorized nigral neurons based on the LC3/FtMt immunofluorescence intensities, degree of colocalization, and subcellular overlaps. As a result, we resolved four patterns (Table 2). The FtMt-IR progression in PSP patients remains unknown because it cannot be assessed in post-mortem tissue and there is no adequate animal model. However, LC3 could help to resolve this issue. In vitro, LC3 progressive accumulation was observed in the process leading to cell death [33]. Similar results were observed in animal models [34]. In a human post-mortem brain, LC3 accumulation correlates with Braak’s stage in Alzheimer’s disease [35]. Therefore, we assumed that the LC3 accumulation level can be used to stage FtMt-IR progression in PSP patients. In this context, pattern 0 may define a normal cell, whereas patterns 1, 2, and 3 may illustrate various stages where cells become more and more damaged (Figure 5, upper pictures).

In conclusion, in the nigral neurons of the PSP patients, concomitant accumulation of LC3/FtMt seemed to be related to mitophagy processes. Using the LC3-IR to stage the progression of neuron damage, we resolved the FtMt-IR progression in the PSP patients. This staging could help to elucidate pathological processes in PSP and to understand the role and function of FtMt in PSP. To illustrate this point, we propose the following hypothesis based on our previous findings (Figure 5). Our previous studies showed that reactive oxygen species (ROS) and inflammatory cytokines, such as TNF-α, IL-1β, and IL-6, induced FtMt expression in the human neuroblastoma cell line IMR-32 [15,36]. ROS and inflammatory cytokines might be involved in the pathophysiology of PSP [37]. All these factors result in cellular stress and the elevation of cell metabolism [38]. In iron-rich cells (such as nigral neurons), this may lead to the excessive production of ROS via Fenton reactions that occur between free cytoplasmic iron and superoxide [39]. FtMt accumulation may protect cells by sequestering iron to reduce intracellular ROS. However, when sustained stress is endured, the FtMt protective function might be overwhelmed, damage to mitochondria accumulates, and the mitophagy process increases, as denoted by LC3 accumulation. Eventually, this leads to cell death [40].

## 4. Materials and Methods

### 4.1. Human Brain Sample

All tissue samples were obtained from the brain bank at the Shiga University of Medical Science. Post-mortem midbrains from normal individuals (n = 4) and patients diagnosed with PSP (n = 4) were prepared as previously described [41]. Briefly, collected samples were fixed in formalin, embedded in paraffin, cut into 5 μm thick sections, and then mounted on glass slides. The clinicopathological features of the patients are shown in Table 3.

### 4.2. Immunohistochemical Localization of FtMt- and LC3-Immunoreactivities

The immunohistochemistry for FtMt was conducted as previously described [20,42,43] with few modifications. Sections were deparaffinized in xylene, rehydrated in ethanol and water, and then washed several times in 10 mM phosphate-buffered saline (PBS, pH 7.4). Deparaffinized sections were blocked with 1% hydrogen peroxide in PBS for 20 min at room temperature (RT) to halt endogenous peroxidase activity. Heat-induced epitope retrieval (HIER) was performed by boiling glass-slide-mounted sections in 1 mM ethylenediaminetetraacetic acid (EDTA) for 4 min using a microwave oven. After several washes with PBS containing 0.3% Triton X-100 (PBST), tissue sections were blocked to reduce non-specific staining with 2% bovine serum albumin (BSA) in PBST for 30 min. The sections were incubated overnight at 4 °C with a mouse monoclonal anti-FtMt antibody (clone C65-2, 2 µg/mL) [43], followed by successive incubations for 1 h at RT with biotinylated anti-mouse IgM BA 2020 (1:500, VectorLabs, Burlingame, CA, USA) and avidin–biotin–peroxidase complex (ABC) (Vectastain ABC Elite kit, 1:3000, Vector Laboratories, Burlingame, CA, USA). PBST was used to wash sections after each incubation and for the dilution of the antibodies and ABC complex. Peroxidase-bound sections were developed for 10 min at RT in 50 mM Tris-HCl (pH 7.6) containing 0.02% 3,3-diamine-benzidine tetrahydrochloride (DAB), 0.3% nickel ammonium sulfate, and 0.005% hydrogen peroxide. Some sections were counterstained with a neutral red stain.

The immunohistochemistry for LC3 was performed mostly as above with the following modifications. HIER was performed just after deparaffinization by boiling glass-slide-mounted sections in 10 mM citrate buffer (pH 6.3) for 4 min. After the HIER, endogenous peroxidase inhibition was performed by incubating sections for 20 min at RT with 3% hydrogen peroxide in PBS. Non-specific staining was significantly reduced by incubating sections in 20 mM HEPES containing 1% BSA and 135 mM NaCl for 5 min at RT. The sections were then incubated with rabbit polyclonal against LC3 (1:1000, MBL, Japan) for 1 h at RT, followed by incubation with HistostarTM (Ms + Rb) (MBL, Nagoya, Japan) for 1 h at RT before revelation with HistostarTM DAB Substrate Solution (MBL, Nagoya, Japan) for 5 min at RT. PBS was used to wash sections after each incubation and for the dilution of the antibody and HistoStarTM reagent. Counterstaining was performed via immersion in hematoxylin for 1 min.

After staining, all glass-slide-mounted sections were extensively washed in water, dehydrated using an ascending ethanol series, cleared in xylene, and coverslipped with Entellan (Merck, Darmstadt, Germany). Digital images of the sections were acquired using an Olympus Microscope BX50 (Olympus, Tokyo, Japan) equipped with a Nikon-D90 digital camera (Nikon, Tokyo, Japan).

### 4.3. Double Immunofluorescence Histochemistry for FtMt and LC3

For the double immunofluorescence histochemistry, the HIER and the blocking step for non-specific staining were the same as described above for the FtMt immunohistochemistry. Sections were incubated overnight at 4 °C with a mouse monoclonal anti-FtMt antibody (2 μg/mL) and rabbit polyclonal against LC3 (1:1000, MBL, Nagoya, Japan). Sections were washed several times with PBS, followed by 1 h incubation with Alexa-Fluor-555-labeled donkey anti-mouse IgG (1:500, Invitrogen, A-31570, Frederick, MD, USA) and Alexa-Fluor-488-labeled goat anti-rabbit IgG (1:500, Invitrogen, ab150077, Frederick, MD, USA). PBST was used to wash the sections after each incubation and for the dilution of antibodies. Following incubations, sections were washed several times with 10 mM PBS. Sections were then incubated with Hoechst 33258 (1:2000, Invitrogen, Frederick, MD, USA) in 0.1 M PBS for 15 min and subsequently washed several times with 0.1 M PBST. True black solution (1:40 diluted with 70% ethanol, Biotium, Fremont, CA, USA) was applied with constant agitation for 50 s to reduce endogenous fluorescence. Sections were extensively washed with PBS and then distilled water. After coverslipping with Immunomount (Thermo Fisher Science, Ann Arbor, MI, USA), digital images of the sections were acquired using a Leica TCS SP8 confocal laser scanning microscope (Leica, Wetzlar, Germany) equipped with a Leica DMi8 microscope. Incubation with a secondary antibody alone did not show unspecific labeling.

### 4.4. Characterization of FtMt and LC3 Colocalization

Colocalization of FtMt and LC3 was determined through the integration of double immunofluorescence images generated from the Leica TCS SP8 confocal laser scanning microscope. The optimal number of z-stacks was determined individually for each sample (20–25 stacks). The resulting images were analyzed using the EzColocalization plugin for the ImageJ image analysis software [44]. The Pearson colocalization coefficient (PCC) for the FtMt and LC3 stacked signal was calculated. The PCC transformed value (z-score) was used to assess the degree of colocalization of FtMt and LC3 in control and PSP cases. Assuming a normal distribution, the z-score was calculated using the formula z = (x − μ)/σ, where x is the test value (PCC) and μ and σ are the mean and standard deviation of all PCC values, respectively. The 3D images in Figure 4 were generated using Imaris 8, which is software that processes microscopy images and enables 3D image visualization, while running on an Intel Xeon E5-1630 v3 processor 64 GB RAM and an AMD Firepro W5100 graphics card. For volume reconstruction at full resolution, TIFF images acquired using a Leica TCS SP8 confocal laser scanning microscope were used. The 3D models were generated using the surface rendering workflow on Imaris 8 (v8.1, Bitplane, Belfast, Northern Ireland, UK) using identical parameters. The surface area detail level (grain size) was set at 0.24 µm with an upper background subtraction that fit into the diameter of the largest sphere (0.904 μm) and the lower background subtraction threshold value set at 10. The colocalization foci were highlighted after 3D model processing using rendering functions in Meshlab software [45] (v2021.07, ISTI-CNR, Pisa, Italy). Briefly, the 3D models created with Imaris were saved in the vrml2.0 format and opened with Meshlab. The cluster decimation algorithm was applied to reduce the number of vertices. Subsequently, the volumes for FtMt-IR were rendered as wireframe (Mesh) and the volumes for LC3-IR were rendered as vertices with “xray” shaders.

### 4.5. Statistical Analysis

Differences between the two unpaired groups were determined using the non-parametric Mann–Whitney U test. Spearman’s rank-order correlation was performed to evaluate the relationships between variables. Statistical significance was set at a *p*-value < 0.05. Values were expressed as mean ± standard error of the mean (SEM). Statistical Package for the Social Sciences (SPSS version 25, IBM, Armonk, NY, USA) was used to perform the statistical analysis.

## Figures and Tables

**Table 1:** Correlation analysis of the FtMt-IR and LC3-IR colocalization in the control and PSP cases.

Group	Pearson Correlation Coefficient (PCC) of the Colocalized Signal	PCC Transformed Value (z-Score)
Control-1	0.539	−0.175
Control-2	0.419	−0.761
Control-3	0.333	−1.181
Control-4	0.339	−1.151
PSP-1	0.655	0.391
PSP-2	0.629	0.264
PSP-3	0.815	1.172
PSP-4	0.87	1.440

**Table 2:** Colocalization features of the LC3-IR over the FtMt-IR based on the colocalization patterns of the 3D-modeled images.

Pattern	FtMt-IR *	LC3-IR *	Colocalization *	Feature
0	−/+, scattered, punctiform	−/+, scattered, punctiform	−/+	Rare colocalization
1	++, numerous, punctiform	+, scattered punctiform	+	Overlapping colocalization
2	++, numerous, punctiform	++, numerous, punctiform	++	Overlapping colocalization
3	+++, dense, packed	++, numerous, punctiform and granular	+++	LC3-IR enclosed in FtMt-IR

* −/+, weak; +, moderate; ++, strong; +++, very strong.

**Table 3:** Clinicopathological data of the individual cases included in this study.

Cases	Age (years)	Gender	Post-Mortem Delay (h)	Clinical Diagnosis
Control-1	60	M	5.0	Pancreatic cancer
Control-2	52	M	10.0	Malignant lymphoma
Control-3	83	F	6.5	Malignant lymphoma
Control-4	64	M	12.0	Prostate cancer/subdural hemorrhage
PSP-1	47	M	<12.0 *	PSP
PSP-2	68	F	5.0	PSP
PSP-3	76	M	10.0	PSP
PSP-4	69	M	13.0	PSP

* Accurate post-mortem delay was not determined. PSP: cases with progressive supranuclear palsy (PSP).

## Data Availability

Not applicable.
